# Longitudinal reliability of Twitter sentiment for measuring mental health and well-being in a UK birth cohort

**DOI:** 10.23889/ijpds.v8i3.2278

**Published:** 2023-09-11

**Authors:** Nina Di Cara, Oliver Davis, Claire Haworth

**Affiliations:** 1 School of Psychological Science, University of Bristol; 2 Bristol Medical School, University of Bristol; 3 The Alan Turing Institute, London

## Introduction & Background

Social media data is increasingly recognised as an important source of behavioural data. It can provide insights into patterns of life and how individuals and groups are feeling. However, many studies into social media’s relationship to mental health and well-being have suffered from poorly developed ground-truth data, which relies on assumed ground-truth labels and data from single timepoints. This means that the accuracy of models at future timepoints cannot be assessed.

Collecting Twitter data from cohorts provides a solution to this issue, given the many years of high quality data that can be used as ground truth. Cohorts can also benefit from the higher-resolution data provided by social media that can supplement their traditional data collection methods.

## Objectives & Approach

We used Twitter data that has been collected with consent from two generations of the Avon Longitudinal Study of Parents and Children (ALSPAC) (N=656). The data is linked to two surveys completed in April-May 2020 and May-July 2020 for validated outcome measures of anxiety, depression, and general well-being.

Using the LIWC and VADER sentiment algorithms, the sentiment categories most highly associated with each outcome were used to develop a multiple regression model for each of anxiety, depression and general well-being using the first survey timepoint. Error from these models in predicting the second timepoint allowed us to assess how well different outcomes are predicted by demographic group.

## Relevance to Digital Footprints

This study illustrates how the collection of digital footprint data can be integrated into existing long-term studies which can be used to provide multiple points of ground-truth data.

## Results

Overall the models for anxiety showed the highest error at the future point. Anxiety in females was predicted more accurately than males, and depression more accurately in males. The older generation was more accurately predicted over all three outcomes.

## Conclusions & Implications

This study has shown that the collection and integration of Twitter data into cohort studies is feasible, and that cohort data provides multiple ground-truth options. This time series data is important for assessing the potential feasibility of mental health inference from online behavioural data, which this study shows may vary across personal characteristics.

In future research we plan to link subsequent surveys from ALSPAC to provide more ground truth time points and explore the temporal stability of predictions, and impacts of model drift on performance.

